# Profiling and assessing the risks of image- and performance-enhancing drugs use during the COVID-19 lockdown

**DOI:** 10.3389/fpubh.2024.1386721

**Published:** 2024-06-19

**Authors:** Ilaria De Luca, Francesco Di Carlo, Julius Burkauskas, Artemisa R. Dores, Irene P. Carvalho, M. Ángeles Gómez-Martínez, Attila Szabo, Hironobu Fujiwara, Cristina Monteiro Barbosa, Marco Di Nicola, Marianna Mazza, Gabriele Sani, Debora Luciani, Mauro Pettorruso, Massimo di Giannantonio, Ilaria Cataldo, Gianluca Esposito, Giovanni Martinotti, Thomas Zandonai, Olivier Rabin, Ornella Corazza

**Affiliations:** ^1^Department of Clinical, Pharmaceutical and Biological Sciences, School of Life and Medical Sciences, University of Hertfordshire, Hatfield, United Kingdom; ^2^Department of Neuroscience, Imaging, and Clinical Science, “G. d’Annunzio” University of Chieti-Pescara, Chieti, Italy; ^3^Laboratory of Behavioural Medicine, Neuroscience Institute, Lithuanian University of Health Sciences, Kaunas, Lithuania; ^4^Laboratory of Neuropsychophysiology, Faculty of Psychology and Education Sciences, University of Porto, Porto, Portugal; ^5^School of Health, Polytechnic of Porto, Porto, Portugal; ^6^Department of Psychology, Pontifical University of Salamanca, Salamanca, Spain; ^7^Institute of Health Promotion and Sport Sciences, ELTE Eötvös Loránd University, Budapest, Hungary; ^8^Institute of Psychology, ELTE Eötvös Loránd University, Budapest, Hungary; ^9^Department of Neuropsychiatry, Graduate School of Medicine, University of Kyoto, Kyoto, Japan; ^10^Decentralized Big Data Team, RIKEN Center for Advanced Intelligence Project, Tokyo, Japan; ^11^The General Research Division, Osaka University Research Center on Ethical, Legal and Social Issues, Osaka, Japan; ^12^Department of Psychometrics, Institute of Psychology, Federal University of Rio de Janeiro, Rio de Janeiro, Brazil; ^13^Department of Geriatrics, Neuroscience and Orthopedics, Institute of Psychiatry and Psychology, Fondazione Policlinico Universitario A. Gemelli IRCCS, Università Cattolica del Sacro Cuore, Rome, Italy; ^14^Department of Psychology and Cognitive Science, University of Trento, Trento, Italy; ^15^World Anti-Doping Agency, Montreal, QC, Canada

**Keywords:** body image, exercise addiction, exercise dependence, pandemic, COVID-19, image and performance enhancing drugs

## Abstract

**Background:**

Image and Performance-Enhancing Drugs (IPEDs) can enhance mental and physical capabilities and impact one’s overall health. Initially confined in sport environments, IPEDs use has become increasingly widespread in a high-performing society. The present study was aimed at profiling IPEDs use during the COVID-19 lockdown among an international sample of young adults.

**Methods:**

A cross-sectional observational study was carried out in eight countries (United Kingdom, Italy, Lithuania, Hungary, Portugal, Spain, Brazil, and Japan) between April and May 2020. The survey questionnaire included validated measurements such as Exercise Addiction Inventory (EAI), Appearance Anxiety Inventory (AAI), and Self-Compassion Scale (SCS) as well as questions about the type of IPEDs, purchasing methods and socio-demographic information.

**Results:**

A total of 736 IPEDs users were included in the survey. Their mean age was 33.05 years (±*SD* = 10.06), and 64.2% were female participants. Overall, 6.8% were found at risk of exercise addiction (EAI >24), 27.6% presented high levels of appearance anxiety, and 24.9% revealed low levels of emotional regulation’s self-compassion. Most participants (55.6%) purchased IPEDs through pharmacies/specialized shops, while 41.3% purchased IPEDs on the Internet. Online IPEDs buyers were mainly men who had higher scores on the Exercise Addiction Inventory. One or more IPEDs classifiable as “potentially risky” were used by 66.3% of the sample. Users of “potentially risky IPEDs” were younger and primarily men. They showed higher scores both on the Exercise Addiction Inventory and Appearance Anxiety Inventory.

**Conclusion:**

This study profiled users of IPEDs when the most restrictive COVID-19 lockdown policies were implemented in all the participating countries. More targeted post-COVID 19 prevention strategies should be implemented according to the emerged socio-demographic and psychopathological traits and cross-cultural differences emerged. Longitudinal studies will also be needed to determine the long-term effect of the COVID-19 lockdown on IPEDs consumption.

## 1 Introduction

On 11 March 2020 the World Health Organization (WHO) declared the start of the coronavirus pandemic, warning about the risks of the SARS-CoV-2 on the respiratory system (1) and on other aspects of the central nervous system, like neural brain connectivity (2, 3). As a result, radical preventive measures were taken to mitigate the risk of contagion affecting the lifestyles of individuals in unprecedented ways (4–6). Such measures also reached the world of fitness, forcing gyms, sports clubs, and swimming pools to close to prevent the virus spread (7). Consequently, people had to adapt their training and eating habits, including the use of a variety of products to improve their athletic and physical performance during the lockdowns (8).

Positive effects have been associated with the performance of physical activity. These positive effects have been attributed to various physiological mechanisms, such as decreased body fat mass, metabolic rate increase, and an increase in cardio-respiratory rate reflected in greater maximal oxygen consumption (9). Recent evidence also suggests that high levels of physical activity can reduce the symptoms of depression, including among the older adult who survived the COVID-19 pandemic (10). On the other hand, although excessive exercise is not considered to be a behavioral addiction in the 5th edition of the Diagnostic and Statistical Manual of Mental Disorders (11), a growing number of studies highlight its potentially harmful physical and mental effects (12–15). Exercise Addiction (EA) is characterized by a strong preoccupation with exercise that might become stereotyped and routine, individuals show significant withdrawal symptoms in the absence of exercise and the preoccupation causes clinically significant distress or impairment in physical, social and occupational areas of functioning (16).

Moreover, excessive exercise has been associated with image- and performance-enhancing drugs (IPEDs) consumption (15, 17, 18).

IPEDs is an umbrella term that encompasses a wide range of compounds used to improve physical performance, lose weight, increase muscle functions and structures, and improve cognitive functions and sexual activity, among others (18). In the world of sports and fitness, anabolic steroids (also known as anabolic-androgenic steroids – AAS) were the most widely used performance-enhancing substances initially. Due to the exponential increase in the use of the Internet and social networks, a wide range of unknown and unregulated substances have spread in the market in recent years. These are often advertised as ‘healthier and safer’ alternatives to common anabolic substances and are publicized by influencers and social network users through very common hashtags such as #fitspiration or #fitspo (19). For these reasons, the IPEDs market is largely uncontrolled (20) and may pose health threats for its users (21, 22). Also, the sale of IPEDs is often supported by captivating marketing strategies and social media advertisements, spreading scientifically unfounded claims and, therefore, raising serious safety concerns (23). In fact, adverse events (especially long-term) are only partially known and a systematic review of the literature revealed that most people using IPEDs, especially smart drugs, are unaware of their risks and potential addiction (24).

Athletes of all categories often use dietary supplements regardless of the decisive aspects of sports performance, represented by constant training, talent, motivation, and tactics. In fact, some individuals often resort even to minimal benefits achieved through the use of supplements (25) and many of these agents may contain undisclosed psychoactive substances (26, 27) such as new psychoactive substances (NPS) (23). This phenomenon is often linked with permissive and unclear legislation (28, 29).

Although some psychological aspects could help to mitigate or deter the use of IPEDs (18), other factors represent a fertile ground for their consumption. Appearance anxiety is described as the fear of being negatively evaluated or rejected by others because of one’s physical appearance (30). People who experience appearance anxiety usually have a negative perception of their body and may engage in risky behaviors to improve their physical and mental health (18, 31). In contrast, self-compassion is associated with psychological well-being and is negatively correlated with shame, regret, and fear of failure that individuals can experience (32). Self-compassion is defined as an emotionally positive self-attitude, characterized by kindness and understanding toward oneself and the possibility to hold painful thoughts and feelings in mindful awareness rather than avoiding them or overidentifying with them (33).

Shibata and colleagues (15) found an unprecedented correlation between exercise addiction, poor self-compassion, and high-level appearance anxiety. Higher levels of exercise addiction and appearance anxiety were positively correlated with a higher tendency to excessive IPEDs use. Higher scores on the Self-Compassion Scale (SCS), acted as a mitigating factor toward excessive exercise and IPEDs use.

During the coronavirus lockdown, individuals might have pursued rewarding behaviors as a coping strategy to deal with the prolonged periods of self-isolation. For example, in research by Dores et al., about half of 564 participants reported a worsening in their mental health due to social distancing, including anxiety and depression (18).

As long as we know, the present study is the first one aimed at profiling those individuals who were most at risk of using IPEDs during the strictest period of the COVID-19 lockdown (April–May 2020) using a cross-cultural sample. The specific objectives of the study is to profile IPEDs users in terms of socio-demographic characteristics and psychological discomfort (compulsive exercising, appearance anxiety, low levels of self-compassion) and/or pre-existing psychiatric disorders: (i) based on the type substance consumed (safe IPEDs vs. risky IPEDs) and (ii) based on purchase method (pharmacies and specialized shops vs. the Internet). This will provide an up-to-date tool for practitioners to better understand and assess the phenomenon of Image- and Performance-Enhancing Drugs intake.

## 2 Methods

### 2.1 Research design

This cross-sectional study consisted of a questionnaire sent via the Web and based on volunteer participation.

### 2.2 Procedure

The research team elaborated the questionnaire and then translated its original English version into six languages (Hungarian, Italian, Japanese, Lithuanian, Portuguese, and Spanish). Subsequently, the questionnaire was translated back into English for the establishment of semantic and conceptual equivalence by the research group. The Qualtrics online research platform (Qualtrics, Provo, UT, 2020) was used for data collection. The survey was disseminated via the Web and using a snowball sampling method, in which participants were invited to fill in the questionnaire and share it with their friends and relatives. Dissemination was also implemented through posts on social media such as Facebook, Twitter, Instagram, LinkedIn, and WhatsApp. Inclusion criteria were (i) age between 18 and 65, (ii) use of at least one IPED, and (ii) consent to participate in the study. The data obtained were securely stored on a password-protected computer at the University of Hertfordshire, Hatfield, United Kingdom (United Kingdom).

Data collection took place during April and May 2020, which was the peak period of lockdown in all nations taking part in the study.

### 2.3 Measures

The survey comprised: (i) sociodemographic questions; (ii) questions on IPEDs use; and (iii) validated psychometric instruments, including the Exercise Addiction Inventory (EAI), Appearance Anxiety Inventory (AAI), and Self-Compassion Scale (SCS- Short Form).

*The EAI* (34) is a tool developed to measure addictive tendencies in exercise behavior. It includes six statements gaging the classic symptoms of addictions (i.e., salience, mood modification, tolerance, withdrawal symptoms, social conflict, and relapse), which are rated on a 5-point Likert scale ranging from 1 (strongly disagree) to 5 (strongly agree). The maximum score obtainable on the EAI is 30. A person scoring 24 or higher may be at risk of exercise addiction. This suggested cut-off score represents the top 15% of the total scale’s score. The EAI is presented as a valid and reliable psychometric instrument, used in many studies across various countries (34–36). Cronbach’s *alpha* in the present sample was 0.641, suggesting an acceptable internal consistency.

*The AAI* (36, 37) is a 10-item tool that measures cognitive and behavioral dimensions of appearance anxiety and symptoms associated with Body Dimorphic Disorder (BDD) (36). In this study, it is rated on a 4-point Likert scale that ranges from 1 (not at all) to 4 (all the time). Values of 21 or more correspond to the top 15% of the total scale’s score. Cronbach’s alpha in the present sample was 0.876, suggesting a very good internal consistency.

*The SCS-Short Form* (38) consists of 12 items and is related to self-compassion and emotional self-regulation. It comprises six subscales: (1) self-kindness, (2) self-judgment, (3) common humanity, (4) isolation, (5) mindfulness, and (6) over-identification. All items are rated on a 5-point Likert scale, from 1 (almost never) to 5 (almost always). A higher score suggests greater self-compassion. Cronbach’s alpha in the present sample was 0.852, suggesting a very good internal consistency.

*IPED questions.* Participants were asked about the forms of supplements or products they used to reach their fitness or physical appearance goals during self-isolation. Further, they were asked about their habitual mode of purchase: pharmacies, specialized shops, or the Internet (web shops). For comparison purposes, the IPEDs listed were the same as those in previous research conducted by Corazza et al. (17). This list was developed in consultation with medical doctors and sports dieticians.

The lead researchers performed an initial data familiarization to generate two IPED categories within the dataset: (1) “non-harmful IPEDs” and (2) “potentially risky (or hazardous) IPEDs.” The extant literature was consulted to refine the two categories and apply them to the full sample (22). The first category includes vitamins, minerals, proteins, amino acids, and natural extracts. The second consists of any other IPED that does not fit in the “non-harmful” category, such as medications, steroids, and stimulants. The list of the IPEDs in the two categories is shown in Table 1.

**Table 1:** List of IPEDs included in the survey.

Variable	Description	Details
Non-harmful IPEDs	Lower risk of side effects, over-the-counter drugs, lower risk in taking without medical supervision, lower potential for abuse	Vitamins, Proteins, Tea or infusions, Multivitamin supplements, Amino acids, Omega 3 fish oil, Multimineral supplements, Mineral salts, Green tea extracts, Antioxidants, Ginseng, Fish oil, Glutamate, Guaran, Turmeric, Herbal medicine, Glucosamine, Beta-alanine
Potentially risky IPEDs	High risk of side effects, mostly prescription drugs, high risk of consumption without medical supervision, high potential for abuse	Nitric oxide, Stimulants (e.g., amphetamine, modafinil), Androgens (e.g., steroids), Various hormones (e.g., EPO, insulin) or related agents (e.g., beta-2 agonists), Diuretics, Glucocorticoids, Ibuprofen, Laxatives, Orlistat, Beta-blockers, Caffeine, Taurine, Creatine, Carnitine, Ketones, Pyruvate

IPEDs, image- and performance-enhancing drugs; EPO, Erythropoietin.

### 2.4 Ethics statement

The Ethics Committee of the University of Hertfordshire, United Kingdom, approved the study (permission: HSK/SF/UH/00104). Also, ethical clearance was obtained from the Ethics Committees of the participating institutions from the various nations. The European General Data Protection Regulation, as well as the norms of the Helsinki Declaration, were rigorously followed during the work (39).

### 2.5 Data analysis

The IBM Statistical Package for Social Sciences (SPSS), developed for the Windows platform, was the software used for data analyses (IBM SPSS Inc., Chicago, Illinois). Basic descriptive statistics consisted of mean, standard deviation, skewness and kurtosis. Only hourly values in the question on ‘time spent online’ were not normally distributed. These values were expressed as median (IQR). The assumptions of normality were not violated for the rest of the analyzed data, which were expressed as means, standard deviations, frequencies, or percentages, as appropriate. Subsamples were compared with Student’s *t*-tests and Mann–Whitney *U* tests for the continuous measures. For categorical data, Chi-square tests and Fisher exact tests were employed. The level of statistical significance was set at *alpha* (*α*) = 0.05.

Based on previous works (15, 18) effect size was estimated to be *d* = 0.25. Thus, with an effect of 0.25, power set at 90% and an *alpha* level of 0.05, the total sample size was calculated to be 735 participants (allocation rate: N2/N1 = 1.8).

## 3 Results

### 3.1 Demographics

There were 736 IPEDs users from Brazil (*n* = 337; 45.7%), Italy (*n* = 134; 18.2%), Spain (*n* = 42; 5.7%), Lithuania (*n* = 78; 10.5%), Portugal (*n* = 51; 6.9%), the United Kingdom (*n* = 36; 4.9%), Japan (*n* = 31; 4.2%), and Hungary (*n* = 27; 3.6%). Their mean age was 33.05 (±SD = 10.06) years, and most were women (*n* = 473; 64.2%).

### 3.2 Psychological measures

Among IPEDs users, 6.8% (*n* = 50) were susceptible to exercise addiction (EAI >24). Another 27.6% (*n* = 203) presented high levels of appearance anxiety (AAI ≥ 21), and another 24.9% (*n* = 183) revealed low emotional regulation-related self-compassion.

### 3.3 Type of IPEDs used

One or more IPEDs in the “potentially risky” category was used by 488 respondents (66.3%). The remaining participants (*n* = 248, 33.7%) used only IPEDs in the “non-harmful” category. Comparisons between users of “potentially risky” and “non-harmful” IPEDs are detailed in Table 2. Users of potentially risky IPEDs were younger (*p* < 0.001) and male participants (*p* < 0.001). They exhibited more elevated scores both on the EAI (*p* < 0.001) and on the AAI (*p* < 0.001). As to cross-cultural comparisons, in Brazil there was a significantly higher use of potentially risky IPEDs (*p* < 0.001), while in Japan the use of non-harmful IPEDs was prevalent (*p* = 0.004). Users of potentially risky IPEDs reported a higher incidence of both psychiatric disorders in general (*p* < 0.001) and of mood disorders other than depression, such as dysthymia, cyclothymia or bipolar disorder (*p* = 0.014). In the group of users of potentially risky (hazardous) IPEDs, there were statistically significantly more smokers (*p* = 0.012). Among those starting the use of IPEDs during the pandemic (*n* = 112; 33.1%), the largest part consumed only non-harmful products (*p* = 0.004). However, among those using IPEDs both before and during the pandemic (*n* = 524; 71.2%), most used potentially risky ones (*p* = 0.003).

**Table 2:** Comparison between participants using only non-harmful IPEDs and those using potentially risky (hazardous) IPEDs.

	Non-harmful	Potentially risky	Statistical test	*p*	*d*
	*n* = 248	*n* = 488			
EAI - *M* ± SD	16.88 ± 3.67	18.11 ± 4.00	−4.044^a^	<0.001	0.32
EAI – *n* (%)			9.314^b^	0.002	
<24	241(97.2%)	445(91.2%)			
≥24	7 (2.8%)	43 (8.8%)			
AAI - *M* ± SD	16.81 ± 4.96	18.85 ± 6.30	−4.813^a^	<0.001	0.35
SCS - *M* ± SD	31.36 ± 5.98	30.50 ± 6.48	1.756^a^	0.080	−0.14
Age - *M* ± SD	35.17 ± 11.44	31.84 ± 9.83	3.901^a^	<0.001	−0.32
Gender – *n* (%)			10.194^b^	0.001	
Male	69 (27.8%)	194 (39.8%)			
Female	179 (72.2%)	294 (60.2%)			
Country – *n* (%)			24.369^b^	0.001	
Lithuania	32 (12.9%)	46 (9.4%)		0.145	
Hungary	11 (4.4%)	16 (3.3%)		0.454	
Spain	19 (7.7%)	23 (4.7%)		0.098	
Italy	52 (21.0%)	82 (16.8%)		0.163	
United Kingdom	13 (5.2%)	23 (4.7%)		0.766	
Portugal	16 (6.5%)	35 (7.2%)		0.725	
Japan	18 (7.3%)	13 (2.7%)*		0.004	
Brazil	87 (35.1%)	250 (51.2%)*		<0.001	
Education - *n* (%)			2.095^b^	0.718	
Secondary	46 (18.5%)	86 (17.6%)			
Bachelor	97 (39.1%)	213 (43.6%)			
Master	75 (30.2%)	137 (28.1%)			
PhD	17 (6.9%)	34 (7.0%)			
Other	13 (5.2%)	18 (3.7%)			
Time on the Internet before the COVID-19 pandemic - *Mdn* (*IQR*)	2.0 (2.0;4.0)	3.0 (2.0;4.0)	58042.5^c^	0.357	0.11
Time on the Internet during the COVID-19 pandemic - *Mdn* (*IQR*)	4.0 (2.63;6.75)	5.0 (3.0;7.0)	55753.0^c^	0.079	0.18
Reported psychiatric disorders - *n* (%)	72 (29.0%)	229 (46.9%)	21.782^b^	<0.001	
Anxiety	55 (76.4%)	188 (82.1%)	1.147^b^	0.284	
Depression	23 (31.9%)	79 (34.5%)	0.159^b^	0.690	
Other mood disorder	5 (6.9%)	44 (19.2%)	6.051^b^	0.014	
Psychosis	0 (0%)	6 (2.6%)	1.925^b^	0.342	
Eating disorder	13 (18.1%)	48 (21.0%)	0.286^b^	0.593	
Personality disorder	1 (1.4%)	7 (3.1%)	0.589^b^	0.685	
Other	4 (5.6%)	15 (6.6%)	0.092^b^	1.000	
Substance addiction *- n* (%)	16 (6.5%)	44 (9.0%)	1.445^b^	0.229	
Smoking - *n* (%)	33 (13.3%)	102 (20.9%)	6.333^b^	0.012	
Alcohol consumption - *n* (%)			6.149^b^	0.188	
Never	46 (18.5%)	82 (16.8%)			
Once a month	76 (30.6%)	116 (23.8%)*		0.047	
2–4 times per month.	86 (34.7%)	189 (38.7%)			
2–3 times per week.	31 (12.5%)	83 (17.0%)			
4 or more times per week.	9 (3.6%)	18 (3.7%)			
IPEDS use - *n* (%)			10.492^b^	0.005	
Started using during the pandemic	51 (20.6%)	61 (12.5%)*		0.004	
We’re not using during the pandemic	38 (15.3%)	62 (12.7%)			
We’re using before and during the pandemic	159 (64.1%)	365 (74.8%)*		0.003	
Have consulted doctor over IPEDs use *- n* (%)	119 (48.0%)	201 (41.3%)	3.010^b^	0.083	

M ± SD, mean ± standard deviation; Mdn (IQR), median (interquartile range); EAI, Exercise Addiction Inventory; AAI, Appearance Anxiety Inventory; SCS, Self-Compassion Scale.^a^Student’s t-test; ^b^Chi-square test or Fisher Exact test; ^c^Mann–Whitney U test. The minimum level of statistical significance was *α* = 0.05.

### 3.4 Image and performance-enhancing drugs purchase methods

Participants were asked about their prevalent source of IPEDs purchase. Most participants (*n* = 409, 55.6%) purchased IPEDs through pharmacies, shops, or specialized shops; 304 (41.3%) purchased IPEDs on the Internet, either on legal sites or on the black market, and 23 participants (3.1%) purchased IPEDs through other modalities.

Participants were compared based on the modality of IPEDs purchase (see Table 3 for detailed information). Participants who purchased IPEDs online were mostly men (*p* < 0.001) and had more elevated EAI scores than did their non-Internet purchasing counterparts (*p* = 0.004). Regarding cross-cultural differences, in Spain and Brazil there was a significantly higher percentage of participants purchasing IPEDs from “pharmacies/ shops/specialized shops. “Potentially risky” IPEDs were purchased online in greater proportions than they were in pharmacies/shops (70.1% vs. 63.3%), in contrast with “non-harmful” IPEDs, which were purchased more frequently in pharmacies or shops (36.7%) than on the Internet (29.9%). The difference, however, failed to reach statistical significance (*p* = 0.060). Although more users have consulted a health professional about the use of IPEDs when buying from pharmacies/shops (46.2%) than from the Internet (40.3%), the difference was not statistically significant (*p* = 0.114). The majority of users consumed IPEDs both before and during the pandemic (*n* = 504, 68.5%). Among those using IPEDs before but not during the pandemic, the majority purchased them from “pharmacies/shops/specialized shops” (*p* = 0.017).

**Table 3:** Comparison between those purchasing IPEDs through pharmacies/shops/specialized shops and those purchasing them on the internet.

	Pharmacies/shops/specialized shops	Internet	Statistical test	*p*	*d*
	*n* = 409	*n* = 304			
EAI - *M* ± SD	17.35 ± 3.84	18.20 ± 3.91	−2.914^a^	0.004	0.22
EAI - *n*(%)			1.512^b^	0.219	
< 24	385 (94.1%)	279 (91.8%)			
≥ 24	24 (5.9%)	25 (8.2%)			
AAI - *M* ± SD	17.99 ± 5.92	18.39 ± 6.01	−0.886^a^	0.376	0.07
SCS - *M* ± SD	31.10 ± 6.43	30.40 ± 6.16	1.459^a^	0.145	−0.11
Age - *M* ± SD	33.20 ± 10.58	32.58 ± 10.42	0.785^a^	0.433	−0.06
Gender - *n*(%)			14.174^b^	<0.001	
Male	123 (30.1%)	133 (43.8%)			
Female	286 (69.9%)	171 (56.3%)			
Country - *n*(%)			27.211^b^	<0.001	
Lithuania	41 (10.0%)	34 (11.2%)		0.606	
Hungary	10 (2.4%)	15 (4.9%)		0.071	
Spain	32 (7.8%)	9 (3.0%)*		0.007	
Italy	66 (16.1%)	65 (21.4%)		0.071	
United Kingdom	10 (2.4%)	23 (7.6%)*		0.001	
Portugal	32 (7.8%)	19 (6.3%)		0.443	
Japan	15 (3.7%)	15 (4.9%)		0.431	
Brazil	203 (49.6%)	124 (40.8%)*		0.020	
Education - *n*(%)			5.521^b^	0.238	
Secondary	68 (16.6%)	63 (20.7%)			
Bachelor	182 (44.5%)	117 (38.5%)			
Master	109 (26.7%)	95 (31.3%)			
PhD	31 (7.6%)	18 (5.9%)			
Other	19 (4.6%)	11 (3.6%)			
Time on the Internet before the COVID-19 pandemic - *Mdn* (*IQR*)	2.5 (2.0;4.0)	3.0 (2.0;4.0)	60578.5^c^	0.553	0.04
Time on the Internet during the COVID-19 pandemic - *Mdn* (*IQR*)	4.50 (3.0;7.0)	5.0 (3.0;6.75)	61301.0^c^	0.749	0.02
Reported psychiatric disorders - *n*(%)	187 (45.7%)	107 (35.2%)	7.970^b^	0.005	
Anxiety	152 (81.3%)	85 (79.4%)	0.148^b^	0.700	
Depression	66 (35.3%)	34 (31.8%)	0.375^b^	0.540	
Other mood disorder	31 (16.6%)	17 (15.9%)	0.024^b^	0.878	
Psychosis	6 (3.2%)	0 (0%)	3.505^b^	0.090	
Eating disorder	41 (21.9%)	19 (17.8%)	0.728^b^	0.394	
Personality disorder	6 (3.2%)	2 (1.9%)	0.461^b^	0.715	
Other	11 (5.9%)	7 (6.5%)	0.052^b^	0.820	
Substance addiction - *n*(%)	30 (7.3%)	26 (8.6%)	0.357^b^	0.550	
Smoking - *n*(%)	64 (15.6%)	65 (21.4%)	3.869^b^	0.049	
Alcohol consumption - *n*(%)			1.414^b^	0.842	
Never	67 (16.4%)	55 (18.1%)			
Once a month	104 (25.4%)	83 (27.3%)			
2–4 times per month.	155 (37.9%)	113 (37.2%)			
2–3 times per week.	67 (16.4%)	44 (14.5%)			
4 or more times per week.	16 (3.9%)	9 (3.0%)			
IPEDS use - *n*(%)			5.926^b^	0.052	
Started using during the pandemic	61 (14.9%)	45 (14.8%)			
We’re not using during the pandemic	64 (15.6%)	29 (9.5%)*		0.017	
We’re using before and during the pandemic	284 (69.4%)	230 (75.7%)			
IPEDs type - *n*(%)			3.541^b^	0.060	
Non-harmful	150 (36.7%)	91 (29.9%)			
Potentially risky	259 (63.3%)	213 (70.1%)			
Have consulted doctor over IPEDs use - *n*(%)	189 (46.2%)	122 (40.3%)	2.502^b^	0.114	

M ± SD, mean ± standard deviation; Mdn (IQR), median (interquartile range); EAI, Exercise Addiction Inventory; AAI, Appearance Anxiety Inventory; SCS, Self-Compassion Scale.^a^Student’s *t*-test; ^b^Chi-square test or Fisher Exact test; ^c^Mann–Whitney U test. The minimum level of statistical significance was *α* = 0.05.

## 4 Discussion

In recent years, there has been an increase in the attention dedicated to the use of IPEDs and NPS in general (40, 41) Since the beginning of the pandemic, a growing number of studies have been conducted to investigate its effects on the use of IPEDs. The COVID-19 breakdown yielded a sudden change in people’s habits and lifestyles. Such a change posed unprecedented risks to overall health and psychophysical well-being. Social distancing and the closure of numerous fitness facilities has affected the use and supply of IPEDs (42). The changes imposed by the pandemic on the use of IPEDs need to be understood, in order to evaluate their long-term impact. The present paper partly addressed this need, profiling the users of IPEDs in a period characterized by great tension and psychological distress through a cross-cultural approach.

Most of our sample already used enhancing substances before the pandemic. At the same time, 15.2% started using them during the lockdown, and 13.6% suspended their use during this period. These supplements were mostly purchased in pharmacies, shops, or specialized shops (55.6%).

Due to the limitations on activities imposed by the COVID-19 pandemic, such as the closure of gyms and dedicated stores, it is likely that social distancing measures would also have a positive impact. By decreasing some of the most prevalent everyday stressors, individuals may be prompted to relax their exercise routines and reduce their compulsive use of IPEDs, which in turn may lead to a decrease in anxiety related to body image. Physical isolation, however, has been found to be more stressful, especially for young adults (18). During confinement, extended exposure to television and online information and certain adverts might also affect mood, image, performance, physical activity, and the use of IPEDs (18).

### 4.1 Purchase method

Some significant differences emerged between individuals who buy IPEDs online versus those who buy them in shops. More men tended to buy these substances on the Internet rather than in specialized shops (43.8% vs. 30.1%; *p* < 0.001). Conversely, more women tended to buy IPEDs in shops rather than on the Internet (69.9% vs. 56.3%). Cross-country comparisons showed that a significantly greater percentage of individuals who preferred to buy IPEDs on the Internet was from the United Kingdom. Conversely, in Spain and Brazil, a significantly greater proportion of users reported purchasing IPEDs from pharmacies or shops. This result is new when compared to previous literature (17, 22), where respondents in the United Kingdom sample predominantly purchased IPEDs also from shops or pharmacies. This practice can be explained at times of lockdown involving social distancing. Still, it could signal a changing trend, which deserves more empirical attention in future works.

Internet buyers scored significantly higher on the EAI, suggesting a higher risk of developing exercise addiction. Recent literature suggests that excessive exercising is positively related to the unsupervised consumption of IPEDs (15, 18), which can be endorsed and facilitated by the online purchase of these substances. Online commercials and false adverts could increase the use of these drugs via misleading marketing strategies that promise physical and mental improvement by promoting them as alternatives to controlled medical or pharmaceutical products (43–45).

### 4.2 Type of substances

The majority of IPEDs users (66.3%) tended to consume potentially risky substances. This group of users of potentially risky IPEDs were mainly younger men who scored higher on the EAI and the AAI. There was no significant association between self-compassion, education level, or time spent on the Internet (both before and during the pandemic), and the use of risky IPEDs.

The group of consumers of potentially risky IPEDs also had a significantly higher incidence of smoking habits and of psychiatric disorders in general (and mood disorders other than depression in particular), when compared with the non-harmful substances group. The most frequently represented psychiatric disorders in the group of users of potentially risky IPEDs (which were the same as in the group of non-risky substance users) were anxiety (82.1%), depression (34.5%), and eating disorders (21.0%). An interesting finding was that individuals who were already using IPEDs before the COVID-19 pandemic, and continued doing so during the pandemic, used significantly more potentially risky substances when compared with both those who stopped taking IPEDs and those who started taking IPEDs during the pandemic. The new users of IPEDs during the pandemic predominantly preferred substances in the “non-risky” category, suggesting that those who initially approach these substances tend to prefer those considered (and advertised) as “safer.” Moreover, they might have started taking supplements during the pandemic as a way to reinforce their immune system against the virus. More individuals taking non-dangerous substances saw a doctor about the consumption of these products (48.0%) than did those taking potentially dangerous substances (41.3%), although the difference was statistically non-significant (*p* = 0.083). Nevertheless, even though negative effects connected with the use of supplements such as creatine, caffeine, and steroids have been broadly documented in the literature (46, 47), the newest IPEDs remain marginally studied and regulated (20, 48). As observed in other studies (49), the consumption of supplements among people who consistently use them is generally perceived as safe, acceptable, and needed for achieving the ideal body form, or weight, and fitness objectives.

This study presents new data on the subject, but also has some limitations. First, the questionnaire was disseminated online and contained self-reported measures, without any biological tests to confirm the data collected on substance use. The second limitation is that it is based on a non-stratified sample of volunteers, which may result in selection bias and, consequently, this sample may not represent the population. For example, it is possible that, because the questionnaire was accessed online by respondents who use the Internet, Internet buyers of IPEDs are overrepresented in the sample (e.g., in the United Kingdom, comparing to previous studies on IPEDs use). Also, the sample sizes by country are quite different, with the Brazilian sample being the largest. Third, within the two groups of (“potentially risky” and “non-harmful”) IPEDs, categories should be broken down further for a better understanding of the tendencies found regarding purchasing risky IPEDs online, and (not) seeing doctors about IPEDs use.

## 5 Conclusion

Previous research has shown a significant correlation between IPEDs use and the likelihood of exercise addiction or body image disorders (13, 17, 18, 23). This work opens up new research scenarios in the field of IPEDs profiling of the most at-risk users at challenging times.

Men most often use hazardous substances and most often purchase them online. Both behaviors correlated with higher levels of physical activity dependence and with smoking habits (although not with substance addiction). The former behavior was also associated with younger ages, appearance anxiety and with a higher prevalence of mental discomfort. The data revealed no significant differences in the amount of time spent on the Internet prior to, and during the pandemic, as well as no differences in self-compassion levels. There was a tendency for potentially risky IPEDs to be acquired online more than in pharmacies/shops (in contrast with non-harmful IPEDs, which were mostly bought in pharmacies/shops), although statistical significance was not reached for this association. In addition, people who purchased on the Internet and used potentially risky IPEDs showed a greater tendency to take them without medical supervision, although this association was statistically non-significant.

Cross-cultural comparisons revealed that, in Brazil, there was a significantly higher use of potentially at-risk IPEDs, while in Japan, the use of non-harmful IPEDs was prevalent. In Spain and Brazil, there was a considerably higher percentage of participants purchasing IPEDs from pharmacies or shops while a higher number of participants from the United Kingdom purchased IPEDs on the Internet.

This work reiterates the importance of a more complete and thorough understanding of IPEDs consumption. Their intake is expected to exponentially grow in the future (50). This gives us the extent of a society increasingly driven toward performativity and achievement, at the expense of physical and mental well-being, and underlines the need of an ethical and social perspective on the consumption physical and mental enhancers (24) It is essential that clinicians and mental health professionals are more aware of the risks associated with IPEDs consumption and possible related psychopathologies or correlated dysfunctional behaviors, both online and offline (23). Identifying an at-risk population has been the first step to facilitate the implementation of evidence-based targeted interventions.

## Data availability statement

The raw data supporting the conclusions of this article will be made available by the authors, without undue reservation.

## Ethics statement

The studies involving humans were approved by The Ethics Committee of the University of Hertfordshire, United Kingdom, approved the study (permission: HSK/SF/UH/00104). Also, ethical clearance was obtained from the Ethics Committees of the participating institutions from the various nations. The European General Data Protection Regulation, as well as the norms of the Helsinki Declaration, were rigorously followed during the work. The studies were conducted in accordance with the local legislation and institutional requirements. The participants provided their written informed consent to participate in this study.

## Author contributions

IL: Writing – original draft, Methodology, Conceptualization. FC: Writing – original draft, Methodology, Conceptualization. JB: Writing – review & editing, Formal analysis, Data curation. AD: Writing – review & editing, Investigation. IPC: Writing – review & editing, Investigation. MG-M: Writing – review & editing, Investigation. AS: Writing – review & editing, Investigation. HF: Writing – review & editing, Investigation. CB: Investigation, Writing – review & editing. MN: Writing – review & editing, Visualization. MM: Writing – review & editing, Visualization. GS: Writing – review & editing, Visualization. DL: Writing – original draft. MP: Writing – review & editing, Visualization. MG: Writing – review & editing, Visualization. IC: Writing – review & editing, Visualization. GE: Writing – review & editing, Visualization. GM: Writing – review & editing, Project administration, Methodology, Conceptualization. TZ: Writing – review & editing. OR: Writing – review & editing. OC: Writing – review & editing, Project administration, Methodology, Conceptualization.
